# Examining Ancient Inter-domain Horizontal Gene Transfer

**Published:** 2008-05-09

**Authors:** Francisca C. Almeida, Magdalena Leszczyniecka, Paul B. Fisher, Rob DeSalle

**Affiliations:** 1 Department of Biology, New York University, New York, NY; 2 Sackler Institute for Comparative Genomics, American Museum of Natural History, 79th Street @ Central Park West, New York 10024, U.S.A; 3 Departments of Pathology, Urology and Neurosurgery, Herbert Irving Comprehensive Caner Center, Columbia University Medical Center, College of Physicians and Surgeons, New York, U.S.A

**Keywords:** horizontal gene transfer, node height test, eschericia coli, blast

## Abstract

Details of the genomic changes that occurred in the ancestors of Eukarya, Archaea and Bacteria are elusive. Ancient interdomain horizontal gene transfer (IDHGT) amongst the ancestors of these three domains has been difficult to detect and analyze because of the extreme degree of divergence of genes in these three domains and because most evidence for such events are poorly supported. In addition, many researchers have suggested that the prevalence of IDHGT events early in the evolution of life would most likely obscure the patterns of divergence of major groups of organisms let alone allow the tracking of horizontal transfer at this level. In order to approach this problem, we mined the *E. coli* genome for genes with distinct paralogs. Using the 1,268 *E. coli* K-12 genes with 40% or higher similarity level to a paralog elsewhere in the *E. coli* genome we detected 95 genes found exclusively in Bacteria and Archaea and 86 genes found in Bacteria and Eukarya. These genes form the basis for our analysis of IDHGT. We also applied a newly developed statistical test (the node height test), to examine the robustness of these inferences and to corroborate the phylogenetically identified cases of ancient IDHGT. Our results suggest that ancient inter domain HGT is restricted to special cases, mostly involving symbiosis in eukaryotes and specific adaptations in prokaryotes. Only three genes in the Bacteria + Eukarya class (Deoxyxylulose-5-phosphate synthase (DXPS), fructose 1,6-phosphate aldolase class II protein and glucosamine-6-phosphate deaminase) and three genes–in the Bacteria + Archaea class (ABC-type FE3+-siderophore transport system, ferrous iron transport protein B, and dipeptide transport protein) showed evidence of ancient IDHGT. However, we conclude that robust estimates of IDHGT will be very difficult to obtain due to the methodological limitations and the extreme sequence saturation of the genes suspected of being involved in IDHGT.

## Introduction

While horizontal gene transfer (HGT) has been widely accepted as an important evolutionary force among prokaryotes (Lawrence and Ochman, 1997; Jain et al. 1999; Ochman et al. 2000), the role of HGT in the early evolution of life has been controversial (Woese, 2002). HGT has been suggested to occur between organisms belonging to the different domains of life: Bacteria, Archaea, and Eukarya (Hilario and Gogarten, 1993; Kandler, 1994, 1998; Gogarten, 1995; Katz, 1996; Aravind et al. 1998; Nelson et al. 1999; Woese, 2002; Klotz and Loewen, 2003). This kind of transfer is quite patent in the numerous cases of mitochondrial and chloroplast genes found in the nuclear genomes of some eukaryotes (Martin et al. 1998; Berg and Kurland, 2000; Rujan and Martin, 2001). Besides the organelle case, however, the importance of ancient inter domain HGT (IDHGT) is still under debate (Teichmann and Mitchison, 1999; Kyripides and Olsen, 1999; Logsdon and Faguy, 1999; Stanhope et al. 2001; Snel et al. 2002; Eisen and Fraser, 2003). It has been suggested that IDHGT was so prevalent in the beginning of life that it would prevent a good assessment of the early branching of the tree of life (Doolittle, 1999a, 1999b). Most evidence for ancient ID HGT, however, is weak and/or based on non-phylogenetic methods that do not support IDHGT versus alternative hypotheses (Kurland, 2000; Koski and Golding, 2001; Koski et al. 2001; Ragan, 2001; Kurland et al. 2003; Brown, 2003). For instance, CG content or distinctive genomic characteristics have been used to suggest HGT in prokaryotes, but these genomic differences could also be due to distinctive evolutionary trends in some lineages related to natural selection (Hayes and Borodovsky, 1998). Another class of tests of HGT, the phyletic distributional profiles based on BLAST searches could also be interpreted as gene loss and are largely affected by the database (Nelson et al. 1999; Salzberg et al. 2001; Roelofs and Van Haastert, 2001; Genereux and Logsdon, 2003).

Here we propose an approach for the detection of HGT and use it to examine ancient classes of IDHGT using phylogenetic analysis—the only available method capable to distinguish HGT from other hypothesis (Logsdon and Faguy, 1999; Ragan, 2001). We also introduce and test a new method based on node height differences in phylogenetic comparisons that is faster than phylogenetic tree searching. Our approach was to make a general assessment of ancient IDHGT using these methods and the available GenBank database. In this way we assess the possibility of detecting reliable evidences of HGT (that can discriminate among alternative explanations for an observed pattern) and possible problems that are often times not taken into account in HGT analyses. In addition, our approach is a first step in examining the ability to detect ancestral IDHGT using robust cladistic methods. By using phylogenetic methods and taking advantage of the most commonly accepted topology of the tree of life, we examine IDHGT between Bacteria, Archaea and Eukarya using the *E. coli* genome as a reference genome.

Since the importance of HGT following endosimbiosis events is well recognized, we focus on HGT that does not involve endosimbiotic associations. Our approach is very conservative and we intentionally do not offer this method as a method to understanding endosymbiotic aspects of inter-domain gene transfer. Several excellent studies have examined the wholesale transfer of genes via endosymbiotic relationships (Karlberg et al. 2000; Palenik, 2002; Martin et al. 2002). Our concern in this paper is to examine those extremely difficult episodes of HGT that did not occur as a result of endosymbiotic relationships. We also do not attempt here to make a thorough search for horizontally transferred genes, since our approach also has several limitations, but the same methodology we propose here could be modified and used in alternative, more thorough analyses. Nevertheless, the approach presented here allows for an estimation of the frequency of HGT events that can be detected within the limitations imposed by the data and the methods available. Our results, using a gram negative bacteria centric analyses indicate that only a few instances of statistically supported evidence of HGT exist. We suggest that this observation is due to substitution saturation and lack of resolution of the phylogenetic trees and that these problems may preclude any good estimation of ancient interdomain transfers.

## Materials and Methods

### Screening for genes with potential for IDHGT

In order to apply phylogenetic methods for detecting HGT, one should be able to produce rooted trees. When the ingroup of the phylogenetic analysis includes all forms of life, the outgroup is usually a paralogous gene and hence our first screening consisted of finding genes with a suitable paralog to be used as outgroup. A list of *E.coli* K-12 paralog genes with 40% or higher similarity level was downloaded from http://www.tigr.org/tigr-scripts/CMR2/LevelsOfParalogy1.spl?db_data_id=99. This list consisted of 1,268 genes from a total of approximately 4,200 genes found in the *E. coli* genome. *E. coli* K-12 was chosen as a guide because it has a well-annotated and fairly large genome among bacteria (Wernegreen et al. 2000).

**Figure 1:**
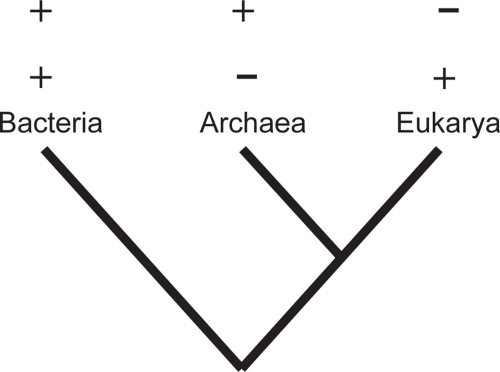
**Distributional profile method**. A first screening for genes involved in ancient IHGT was done using the distributional profile method. Based on the most accepted hypothesis of phylogenetic relationships between the three domains of life, genes that occur in Eukarya and Bacteria, but not in Archaea, or genes that occur in Archaea and Bacteria, but not in Eukarya were potential candidates to have been horizontally transferred between domains. Genes found in one of those two phyletic distributional categories were further tested for HGT (see text).

Our second step in the screening was to look for genes that have a taxonomic distribution in the three domains that deviates from the expected. This screen was based on the most accepted hypothesis for the tree of life that suggests a closer relationship of Archaea and Eukarya to the exclusion of Bacteria (Searcy et al. 1978; Zillig et al. 1989, 1992; Iwabe et al. 1989; Gogarten et al. 1989; Brown et al. 2001). Using a phyletic distributional profile with the specific inter-domain distributions boxed in Figure 1 as a guide, we focused on orthologs that exist in Bacteria AND either Archaea OR Eukarya. Therefore among the 1,268 genes we examined, we looked for those that were present in Archaea but absent in Eukarya, and those that were present in Eukarya but absent in Archaea. Based on the (Bacteria(Archaea, Eukarya)) hypothesis, there are two alternative explanations for these distributional profiles: the gene was either present in the universal common ancestor and posteriorly lost in the domain that lacks it, or the gene was horizontally transferred after the split of the domains.

To obtain the distributional profile of the genes in the first list, we used BLAST (blastp) searches against the all the available data in the GenBank at the time of the searches. To make the taxonomic screen of the paralogs easier, we conducted these searches using the “Blink” option, which shows the results of the search color-coded by taxonomic group: Archaea, Bacteria, and Eukaryotes subdivided into Metazoa, Plants, Fungi, and other Eukaryotes. The “Blink” is a link available for each sequence on the NCBI website. Because the “Blink” gives only the 200 best hits, when this number was reached with hits for the same gene, the distribution was double-checked using Blastp to confirm absence of the gene in Archaea and Eukarya. In this case, we used e^−10^ as a cut off value for the presence of a gene in a given domain. To be useful as an outgroup, a paralog should have appeared in a duplication event that occurred before the split of the three domains, instead of being exclusive to Bacteria. Hence, we used the “Blink” as described above to check the distribution of the paralogs. Only genes with a paralog that seem to have appeared before the split of the three domains were retained for further analyses. We also discarded from the analyses all the genes for which orthology and paralogy could not be promptly and confidently determined, and the genes that are known to be involved in mitochondrial or chloroplast metabolism. Amino acid sequences of the genes included in the analyses and their paralogs were downloaded and aligned with ClustalX using standard parameters.

### Phylogenetic analysis

Maximum parsimony trees were obtained with PAUP 4.01 (Swofford, 2003) using heuristic search with 10 random stepwise additions. Analyses were done using at least five paralog sequences. Full bootstrap tests with 500 replicates were performed to test consistency of the branches of strict consensus trees. Evidence of HGT from bacteria to eukaryotes (or archaeans) would be illustrated by a paraphyly of bacteria, with some of them more closely related to eukaryotes or archaeans than to other bacteria. Our focus on parsimony approaches is reasonable and conservative. In fact, we avoid making an inference about a HGT when we detect saturation of sequence changes in our tests.

### Node height test

The node height test, like the phylogenetic analysis, has the objective to test the different hypotheses of lineage extinction (gene loss in a determined lineage) and HGT. Other approaches using rates of evolution to test for HGT have been discussed in Novichkov et al. (2004). The test we present here compares substitution rates within and among groups (here among domains). In the case of lineage extinction, it is expected that the substitution rates within groups will be higher than the rates among groups (see Fig. 3 for a graphical explanation). In the case of HGT, however, there should be no differences in the average substitution rates within and among groups. The test is dependable on homogeneous substitution rates across taxa. Homogeneity of substitution rates across domains was tested with the software RRTree (Robinson et al. 1998). The rate test was done on the whole sequence alignment for all genes. For the genes saturated with substitutions, the regions with gaps and poor alignment were trimmed and the test was redone on the remaining residues. The node height test was performed on the genes for which it was possible to rule out substitution rate differences. A pair wise distance matrix was obtained with PAUP* 4.01b (Swofford, 2002) for each of those genes. Average distances within Bacteria (B1-B1) and between bacteria and the other domain in which the gene is present (B1-E1 or B1-A1) were compared with ANOVA. A node height effect was detected when B1-E1 or B1-A1 was not significantly larger than B1-B1(Supplemental Material File 1).

## Results and Discussion

### Phyletic distributions of genes across domains

Our first screen was based on the most accepted hypothesis for the tree of life that suggests a closer relationship of Archaea and Eukarya to the exclusion of Bacteria (Iwabe et al. 1989; Brown et al. 2001) as described above. Among the 1,268 genes we examined, 402 were present in all three domains, 545 were present only in Bacteria, 95 were also present in Archaea but absent in Eukarya, and 86 were present in Eukarya but absent in Archaea. For 140 genes it was not possible to determine if the hits obtained in the BLAST searches were orthologs or paralogs, mostly due to nomenclatural problems. These categories of genes indicate some evolutionary discontinuity if the sister pair Archaea and Eukarya do not have the same distribution pattern. We first examined if the patterns obtained using phylogenetic analysis are consistent with HGT or lineage extinction.

### Bacteria—Eukarya exclusive patterns

Among the genes present only in Bacteria and Eukarya, 21 were involved in mitochondrial or plastid metabolism, including those physically localized either in the nuclear or organellar genome. The horizontal transfer of those genes is not under question and we excluded them from further analyses. Twenty-six genes were also discarded for having spotty distributions in the three domains, and 19 for problems with orthology and paralogy determination or absence of a useful paralog (see Supplemental Materials File 2 for list of genes and taxa examined). Twenty genes were tested and for three of them the phylogenetic analysis suggests ancient IDHGT from bacteria to eukaryotes (Table 1). Lack of statistical support prevented us from rejecting IDHGT or gene loss for the remaining genes. All the 3 genes recovered here as probable IDHGT cases had already been described as such. Deoxyxylulose-5-phosphate synthase (DXPS) is an enzyme that participates in several pathways involving coenzyme and carbohydrate metabolism. It is the first enzyme of an alternative pathway for the production of isoprenoids and is present only in bacteria and plants (Lange et al. 2000). In our analysis DXPS clusters with proteobacteria with a high bootstrap support (Supplemental Material File 3). This group of Bacteria includes plant-symbiotic species and the ancestor of mitochondria, which may place this gene in the general category of organelle to nucleus HGT.

The second protein, fructose 1,6-phosphate aldolase class II is involved in sugar metabolisms and participates in the glycolysis I and gluconeogenesis pathways. The study of this enzyme is complicated by the presence of several types that represent distinct paralogs (Sanchez et al. 2002). The fructose 1,6-phosphate aldolase class II protein is found in fungi and protists, but those two groups do not form a clade in our analysis (Supplemental Materials File 3).

**Figure 2:**
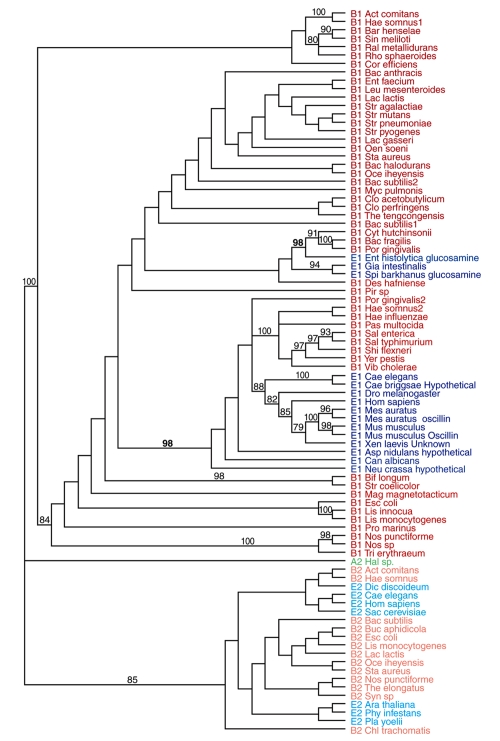
**Maximum parsimony analysis of the gene Glucosamine-6-phosphate (*nagB*, COG0363)**. The tree depicts the strict consensus of 6 most parsimonious reconstructions. Bootstrap values above 75 are shown on nodes. Rooting was done with the paralog gene Phosphogluconate. E stands for Eukarya, B for Bacteria, and A for Archaea. The number 1 refers to sequences of the gene under analysis, and the number 2 to sequences of the paralog used for rooting.

The third protein, glucosamine-6-phosphate deaminase, is also involved in sugar metabolism and participates in the pentose phosphate and Entner-Doudoroff pathways. Andersson et al. (2003) had already reported this gene as a case of HGT, but the lack of rooting precluded an interpretation of the direction of the transfer by these authors. The tree presented here clearly supports an HGT from eukayotes in the lineage of animals and fungi to proteobacteria (Fig. 2). Our analysis also indicated a transfer involving the protist *Entamoeba histolytica* and a group of bacteria, as suggested by Andersson et al. (2003). However, in disagreement with their results, all protists clustered in the same clade and it seems more likely that transfer occurred from the protist to the bacteria.

### Bacteria—Archaea exclusive patterns

The analysis of the genes present only in Archaea and Bacteria showed that three genes were probably transferred from the former domain to thermophilic bacteria: ABC-type FE3+-siderophore transport system, ferrous iron transport protein B, and dipeptide transport protein (Table 2, Supplemental Materials File 3). All 3 genes are involved in cellular transport and two of them specifically in iron transport. HGT from Archaea to thermophilic bacteria has been reported to be as high as 24%, but this percentage was obtained based only on the overall similarity of the genomes and most of the phylogenetic tests we performed failed to support HGT (Nelson et al. 1999; Logsdon and Faguy, 1999). Although we included many different species of thermophilic bacteria, only the ones belonging to the genus *Thermotoga* were found to be involved in the cases of possible HGT. The Thermotogales are believed to be the most basal group of bacteria and the grouping of their genes with those of archaeans may be due to retention of ancestral sequences under strong selection on particular genes in a hot, harsh environment (Kyripides and Olsen, 1999; Logsdon and Faguy, 1999). Even convergence cannot be ruled out.

**Figure 3:**
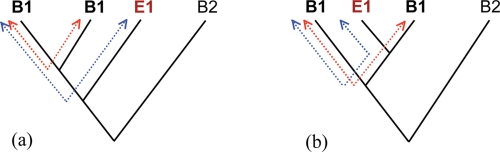
**The node height test.** (**a**) If an Euk-Bac distribution was caused by gene loss, than the E1-B1 (Eukarya to Bacteria) average distance is expected to be higher than the average B1-B1 (Bacteria to Bacteria) distance. (**b**) However, if eukaryotic genes (E1) were gained by transfer from a particular group of bacteria (B1), than the average E1-B1 distance should not be higher than the average B1-B1 distance. These predictions were done based on the assumption that substitution rates are homogeneous across taxa.

A more careful analysis of the candidate genes for HGT between those taxa is still needed to provide better support for HGT. Phylogenetic analysis suggested HGT for 3 other archaeal genes, formate dehydrogenase (cytochrome B556 subunit), glucose-1-phosphate thymidylyltransferase, and adenine deaminase, but the direction is not clear (Supplemental Material File 3). For all other genes the phylogenetic analyses gave very poor resolution and was unable to recover both Bacteria and Archaea as monophyletic groups. However, for one gene, protein secretion membrane protein, HGT was statistically rejected and gene loss is clearly the most likely hypothesis (the phylogenetic tree obtained for this gene resembles the one shown in Fig. 3a).

### The node height approach to HGT detection

Here we introduce and show the results of an alternative test that we propose discerns between HGT and Lineage Extinction called the node height test. Since differences in substitution rates may give a similar effect, the test can be applied only to genes that have homogeneous substitution rates across the different domains. Tables 1 and 2 show the results of the rate test for the bacterial-eukaryotic exclusive genes and bacterial-archaeal exclusive genes, respectively. Most of the genes were saturated with substitutions and could not be tested using the node height test. Nevertheless, a positive effect was detected for both DPXS and fructose 1,6-bisphosphate aldolase class II (protists only), in agreement with the results of phylogenetic analysis. The test was not done on the glucosamine-6-phosphate deaminase gene because it was saturated.

Among the bacterial-archaeal specific genes, a larger number were saturated and could not be tested (Table 2). A node height effect was detected for adenine deaminase and dipeptide transport. However, the effect detected for each gene suggest opposite directions of HGT (Bacteria to Archaea, and Archaea to Bacteria, respectively), corroborating the results of the phylogenetic analyses.

### IDHGT estimation

We found 3 genes that were involved in HGT between Bacteria and Eukarya and 6 genes that were involved in HGT between Bacteria and Archaea. The results obtained here were expected in view that IDHGT is for obvious biological reasons more likely to occur between simple, unicellular organisms. No instance of HGT involving animals was detected. Although the results corroborate the importance of IDHGT for some taxa, it suggests that first, IDHGT is not so prevalent as suggested before and second that it is mostly restricted to some groups.

We acknowledge that the approach used here restricted the number of genes that could be tested. Some of these limitations were imposed by the methodology itself, but some are related to the data.

In the first case, for instance, we did not include genes that are present in the three domains, even though these genes could also be involved in IDHGT. It could happen that one gene was present in the universal ancestral, lost in one of the domains, or some species of one of the domains, and reacquired through IDHGT. That includes the 402 genes found in all three domains. Nevertheless, the approach, including the Node Height test, can be easily automated and could be used in highthroughput screens. We were also limited by the starting dataset: genes present in *E. coli*. Although this species has a fairly large genome among Bacteria, it lacks many genes that are present in other bacterial groups, for instance the gram positive Bacteria. In fact, some gram positive bacterial genes and Archaeal genes appear to be more similar to each other (Brown et al. 1994; Benachenhou et al. 1993) suggesting a focus on gram positive or Archaeal genomes might reveal even more cases of IDHGT. The approach used here could easily be adapted to search for these genes using the genome of a gram positive Bacteria as a starting point, replacing the *E. coli* genome. In addition, our dataset was limited by our cutoff for paralogs. However, given the fact that many of the paralagous gene families we examined at the 40% cutoff showed saturation, we suggest that an even lower cutoff would have resulted in even more extreme saturation. Limitations due to the data include sequence availability, substitution saturation in the sequences, lack of paralogs, and nomenclatural problems. Many genes had to be discarded for the latter reason, indicating the urge to find a better way to name and classify genes.

### Are there acceptable methods of HGT detection?

The node height test corroborated the results of the phylogenetic analysis in almost all the cases we detected and may be used alternatively in cases where the taxa are well represented, since it is less time consuming. The weakness of this test is its dependence on homogeneous substitution rates, which makes it useful for testing only a limited number of genes. Yet, the problem with saturated substitution is likely to affect the results of phylogenetic analysis in a similar way, producing poorly resolved trees and lack of statistical support for nodes. The correct identification of orthologs and paralogs is crucial for both methods. It is important to keep in mind that phylogenetic analysis, despite being the most reliable method, can also give false evidence of HGT in cases of convergence, retention of ancestral character states, and higher evolutionary rate in one particular lineage (long-branch attraction). All those problems are more likely to occur in highly divergent genes that are saturated with substitutions, as is the case of most of the genes studied here. Hence, limitations of the methods may preclude a good estimate of inter-domain HGT and decrease considerably, the robustness of inferences concerning IDHGT. Our results suggest that ancient inter domain HGT is restricted to special cases, mostly involving symbiosis in eukaryotes, specific adaptations in prokaryotes, and specific cases in single celled eukaryotes (Doolittle, 1998; Andersson et al. 2003; Huang et al. 2004; Huang et al. 2004; Andersson et al. 2005; Huang et al. 2005; Huang and Gogarten, 2006).

## Conclusion

We present a new method for detection of HGT that corroborates most of the results obtained with phylogenetic analysis. It is important to note that we limited our study to just those genes in the *E. coli* genome that have orthologs, but we suggest this gene set is a good first approximation of the utility of the approach. The node height test although restrictive was reliable, corroborating the results of the phylogenetic analyses. Since this method is quick and easily automated, it could be used in automates screens for HGT including a larger dataset than the one analyzed here. The grand majority of the genes that by our method are suggested to be involved in ID HGT have also been indicated in other studies that use phylogenetic methods. Although our approach is very limited, this result suggests that most HGT cases that can be identified using phylogenetic methods have already been identified and are in agreement with previous claims that the occurrence of HGT has been overestimated and alternative hypothesis largely put aside (Kyripides and Olsen, 1999; Kurland et al. 2003; Genereux et al. 2003). In conclusion, we suggest that in general reliable estimates of ancient IDHGT will be very difficult to obtain due to methodological and data limitations as discussed above.

## Supplementary Material

File 1— The node height test This file contains a figure detailing the steps involved in the node height test. a) The first step concerns a rate test where substitution rates are examined and their similarity determined. b) If rates are not similar across the tree or there are heterogeneous substitution rates among domains the node height test should not be used; c) If (E1-B1) > (B1-B1) then lineage extinction in Archaea can be inferred. If (A1-B1) > (B1-B1), then lineage extinction in Eukarya can be inferred. d) If (E1-B1)≈(B1-B1) then ancient IDHGT can be inferred between Eukarya and Bacteria. If (A1-B1)≈(B1-B1) then ancient IDHGT can be inferred between Archaea and Bacteria. The bottom of the figure shows a Flow chart outlining the steps in the Node Height Test.

File 2— List of genes examined in study This file contains the list of genes from the *E. coli* genome that have paralogs with 40% similarity elsewhere in the genome. Column 1 indicates the distribution of the gene in other organisms where 1 = bacteria only; 2 = all three domains; 3 = Bacteria and Archaea 4 = Bacteria and Eukarya; 5 = No inference. Column 2 shows the gene name, column 3 shows the most similar paralog, column 4 shows the E value for the comparison of paralogs, column 5, and column 6 indicate simillarities.

File 3— Cladograms that support inter domain HGT This file contains cladograms obtained for genes with phylogenetic evidence of IDHGT. Trees are strict consensus cladograms and numbers shown are bootstrap values above 75 (500 reps). Bootstrap values in bold font indicate possible HGT events. (a) 1-Deoxyxylulose-5-phosphate synthase (*dxs*, COG3959/3958), paralog Transketolase 2 isozyme; (b) Fructose-bisphosphate aldolase, class II (*fba*, COG0191), paralog Tagatose-bisphosphate aldolase 1; (c) Dipetide transport protein (*dppa*, COG0747), paralog Putative transport periplasmic protein; (d) ABC-type FE3+-siderophore transport system, permease component (*HemU*, COG0609), paralog ABC-type FE3+-siderophore transport system, ATP-binding; (e) Ferrous iron transport protein B (*feoB*, COG0370), paralog GTP-binding protein; (f) Formate dehydrogenase (*fdhF*, COG0243), paralog Nitrate reductase 1; (g) Glucose-1-phosphate thymidylyltransferase (*rmla*, COG1213), paralog Glucose-1-phosphate adenylyltransferase; (h) Adenine deaminase *adeC*, COG1001), paralog Putative N-acetylgalactosamine-6-phosphate deacetylase. To facilitate viewing the figures we have colored taxa from the three major domains as follows: Bacteria target genes (B1) are colored red; Bacteria paralog genes (B2) are colored orange; Archaea target genes A1) are colored green; Archaea paralog genes (A2) are colored lime; Eukarya target genes (E1) are colored blue; Eukarya paralog genes (E2) are colored aqua.

## Figures and Tables

**Table 1:** Summary of results for genes with a Bacteria—Archaea exclusive pattern. Results of the relative rate test, rate test using only conserved gene regions, node height test, and support of HGT given by phylogenetic analyses.

Gene name	Rate test	Rate test cons.	Node height test	Trees
2-octaprenyl-6-methoxyphenol	k.s.	k.s.	-	no
ATP-dependent specificity component of clpP serine protease, chaperone	k.s.	k.s.	-	no
glucosamine-6-phosphate deaminase	k.s.	k.s.	-	yes
GTP-binding elongation factor, may be inner membrane protein	k.s.	k.s.	-	no
poly(A) polymerase I	k.s.	k.s.	-	no
putative GTP-binding factor	k.s.	k.s.	-	no
guanylate kinase	k.s.	*	-	no
3-demethylubiquinone-9 3-methyltransferase	k.s.	n.s.	**	no
biosynthetic arginine decarboxylase	k.s.	n.s.	**	no
esterase D	k.s.	n.s.	**	no
glyoxylate-induced protein	k.s.	n.s.	**	no
heat shock protein hslVU, ATPase subunit, homologous to chaperones	k.s.	n.s.	**	no
probable protein-tyrosine-phosphatase	k.s.	n.s.	**	no
pyridoxal kinase 2/pyridoxine kinase	k.s.	n.s.	**	no
enoyl-[acyl-carrier-protein] reductase (NADH)	k.s.	n.s.	**	no
glutathionine S-transferase	n.s.		**	no
polynucleotide phosphorylase	n.s.		**	no
probable 6-phospho-beta-glucosidase	n.s.		**	no
1-deoxyxylulose-5-phosphate synthase	n.s.		n.s.	yes
fructose-bisphosphate aldolase, class II	n.s.		n.s.	yes

* **Notes:** Significant at α 0.05,

** Significant at α 0.01.

**Abbreviations:** k.s.: substitution saturation; n.s.: non-significant at α 0.05.

**Table 2:** Summary of results for genes with a Bacteria—Eukarya exclusive pattern. Results of the relative rate test, rate test using only conserved gene regions, node height test, and support of HGT given by phylogenetic analyses.

Gene name	Rate test	Rate test cons.	Node height test	Phylogenetic analysis
ABC-type FE3+-siderophore transport system, permease component	k.s.	ks.	-	yes
ATPase of high-affinity potassium transport system, B chain	k.s.	k.s.	-	no
excision nuclease subunit A	k.s.	k.s.	-	no
ferrous iron transport protein B	k.s.	k.s.	-	yes
glucose-1-phosphate thymidylyltransferase	k.s.	k.s.	-	yes
phosphoheptose isomerase	k.s.	k.s.	-	no
pleiotrophic effects on 3 hydrogenase isozymes (HypD)	k.s.	k.s.	-	no
protein secretion, membrane protein	k.s.	k.s.	-	no
suppresses inhibitory activity of CsrA	k.s.	k.s.	-	no
thiamin-monophosphate kinase	k.s.	k.s.	-	no
transcriptional regulatory protein (HypAF)	k.s.	k.s.	-	no
transport of potassium	k.s.	k.s.	-	no
FKBP-type peptidyl-prolyl cis-trans isomerase	k.s.	k.s.	-	no
plays structural role in maturation of all 3 hydrogenases (HypE)	k.s.	k.s.	-	no
site-specific recombinase, acts on cer sequence of ColE1, effects chromosome segregation at cell division	k.s.	*	-	no
4-aminobutyrate aminotransferase	k.s.	n.s.	**	no
dipeptide transport protein	k.s.	n.s.	n.s	yes
formate dehydrogenase, cytochrome B556 (FDO) subunit	k.s.	n.s.	**	yes
glucose-1-phosphate uridylyltransferase	k.s.	n.s.	**	no
high-affinity phosphate-specific transport system	k.s.	n.s.	**	no
part of maltose permease, inner membrane	k.s.	n.s.	**	no
phosphotransacetylase	k.s.	n.s.	**	no
regulator for asnA, asnC and gidA	k.s.	n.s.	**	no
spermidine/putrescine transport system permease	k.s.	n.s.	**	no
UDP-N-acetyl-D-mannosaminuronic acid dehydrogenase	k.s.	n.s.	**	no
probable adenine deaminase (synthesis xanthine)	k.s.	n.s.	n.s.	yes

* **Notes:** significant at α 0.05

** significant at α 0.01.

**Abbreviations:** k.s.: substitution saturation; n.s.: non-significant at α 0.05.
