# Supplementary material for: Examining Ancient Inter-domain Horizontal Gene Transfer
Source: Evol Bioinform Online. 2008 May 9;4:109–19. (PMC2614185)
Supplement: File 1— — This file contains a figure detailing the steps involved in the node height test. a) The first step concerns a rate test where substitution rates are examined and their similarity determined. b) If rates are not similar across the tree or there are heterogeneous substitution rates among domains the node height test should not be used; c) If (E1-B1) > (B1-B1) then lineage extinction in Archaea can be inferred. If (A1-B1) > (B1-B1), then lineage extinction in Eukarya can be inferred. d) If (E1-B1)≈(B1-B1) then ancient IDHGT can be inferred between Eukarya and Bacteria. If (A1-B1)≈(B1-B1) then ancient IDHGT can be inferred between Archaea and Bacteria. The bottom of the figure shows a Flow chart outlining the steps in the Node Height Test. [file ebo-4-109-s1.pdf]

Supplemental Material File 1.

Trees explaining Node Height Test

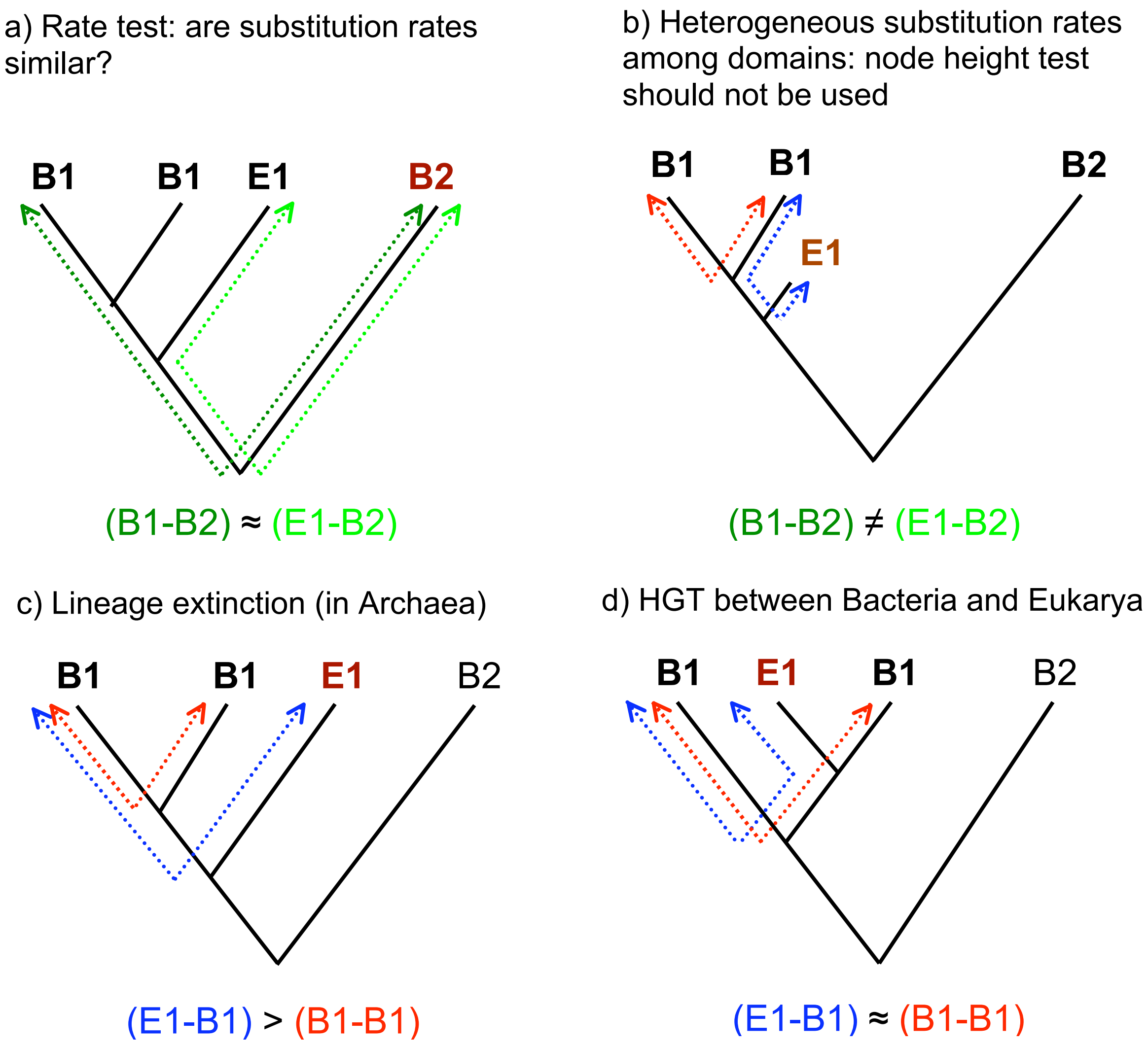

Flow chart showing decision making process in Node Height Test

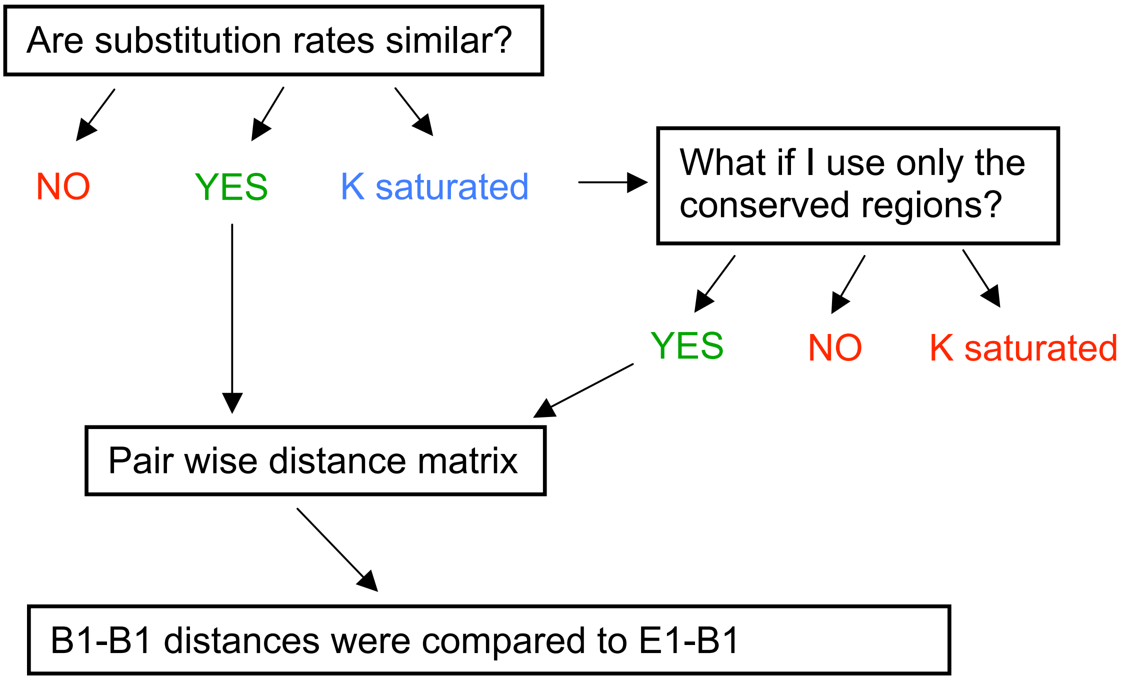

(E1-B1) ≈ (B1-B1)
