# Supplementary figures and images for: Examining Ancient Inter-domain Horizontal Gene Transfer
Source: Evol Bioinform Online. 2008 May 9;4:109–19. (PMC2614185)

(a)

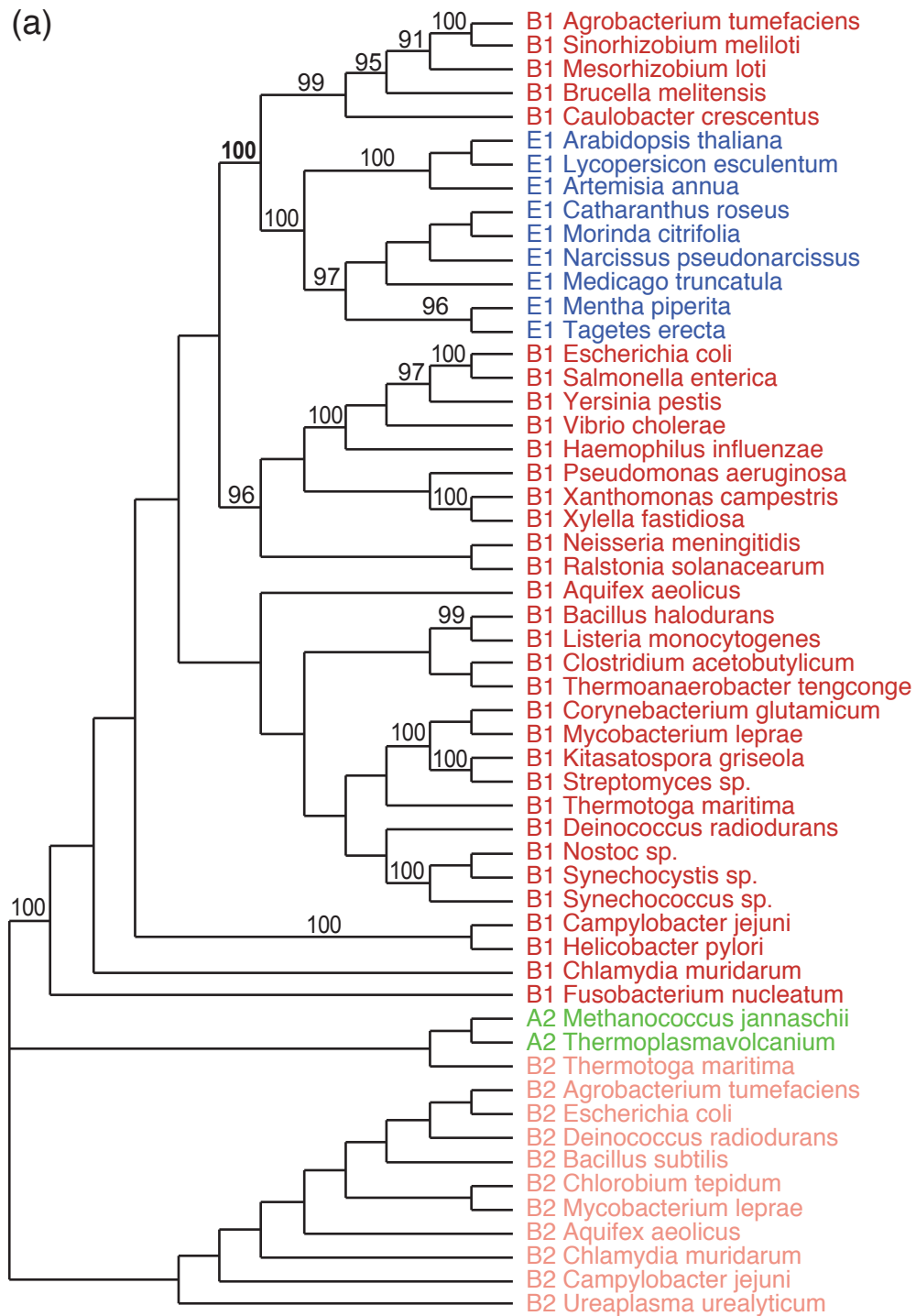

(b)

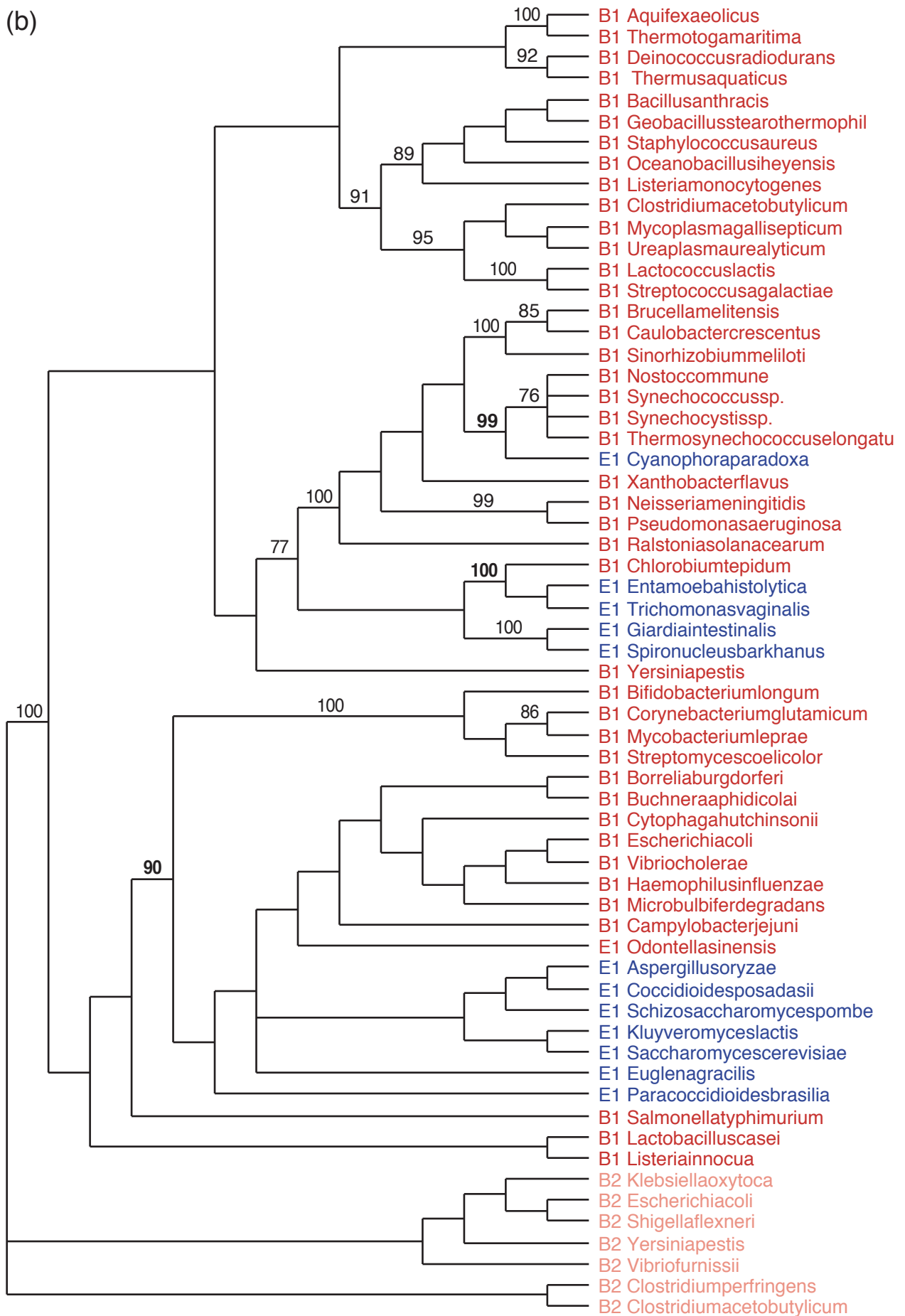

(c)

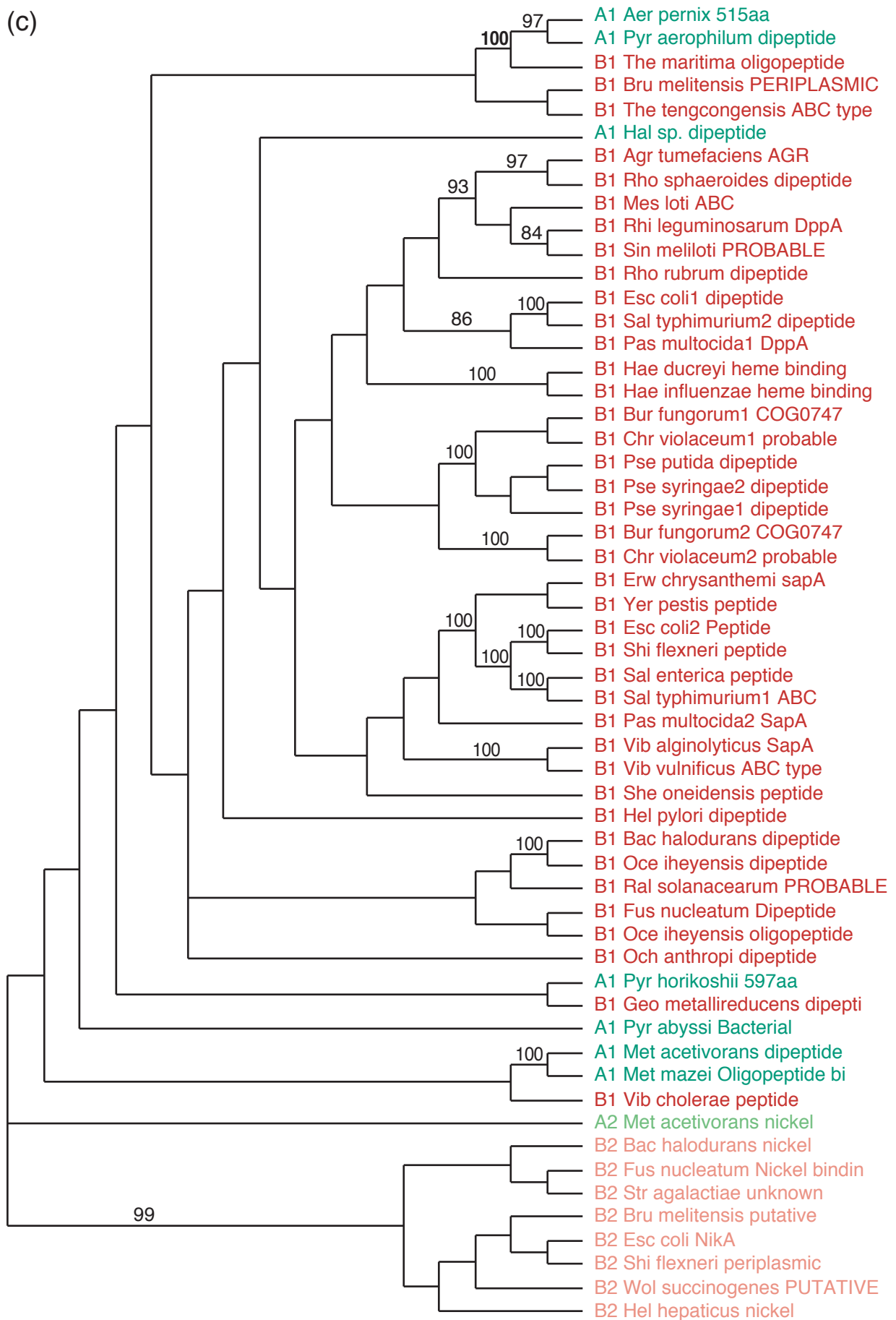

(d)

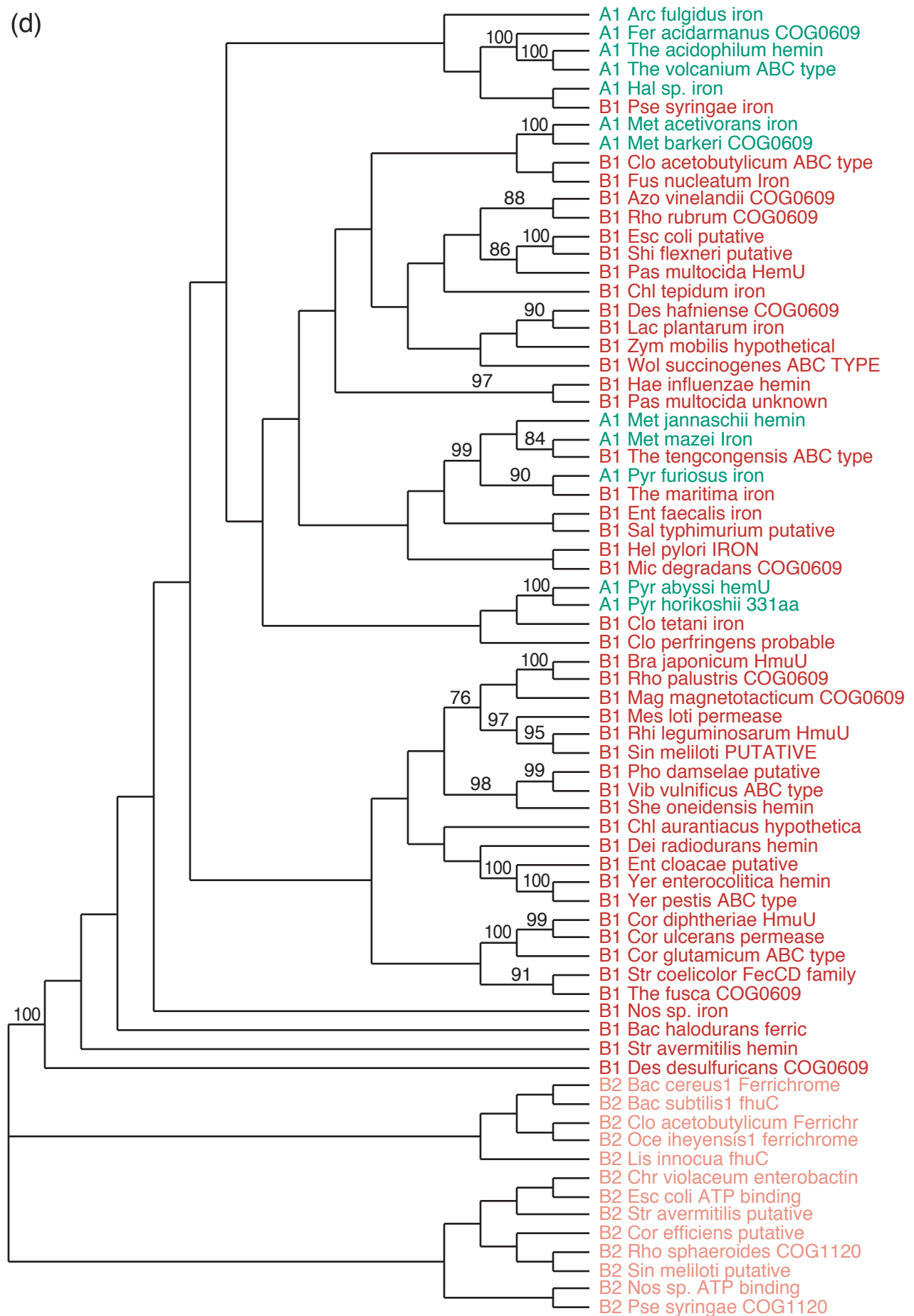

(e)

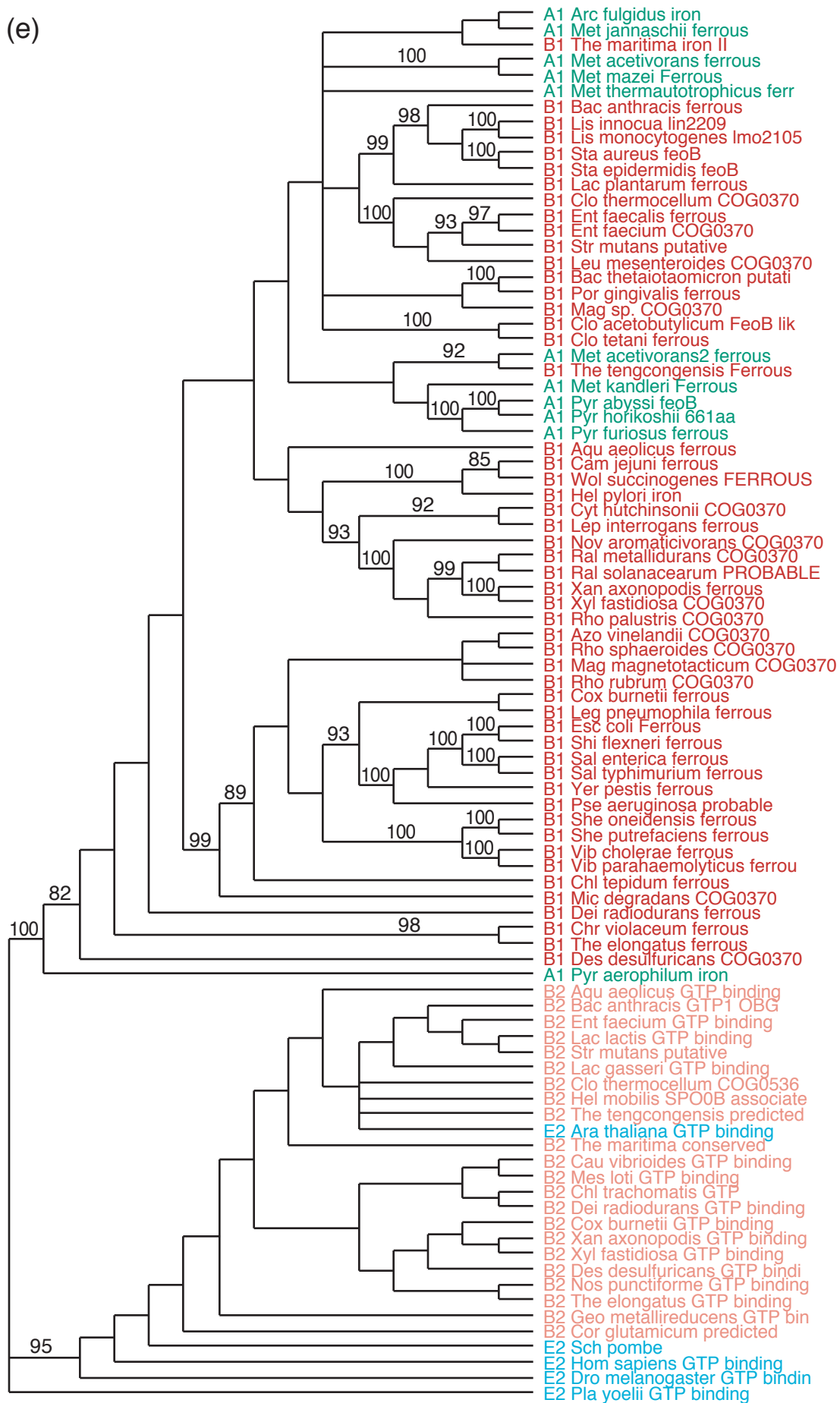

(f)

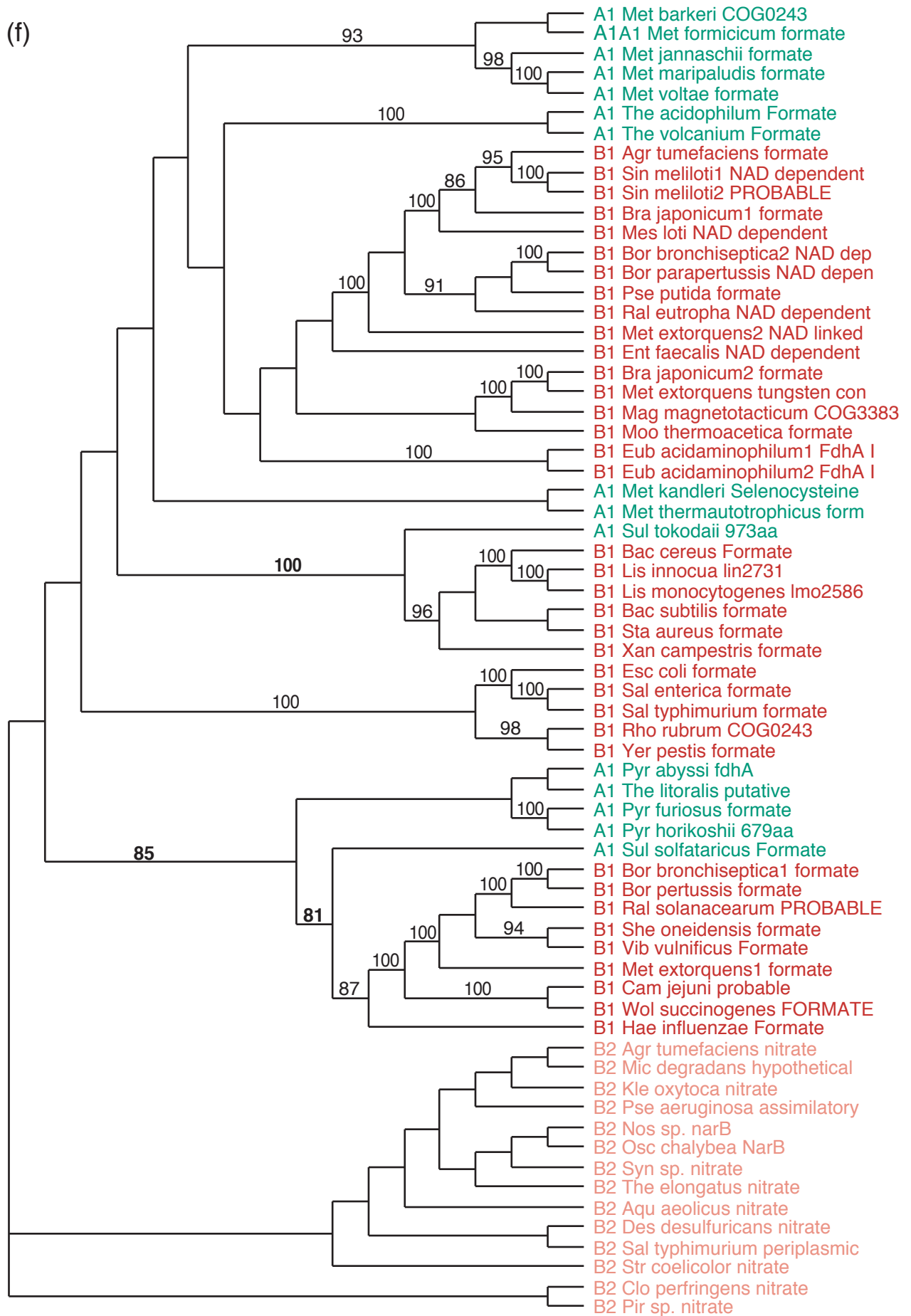

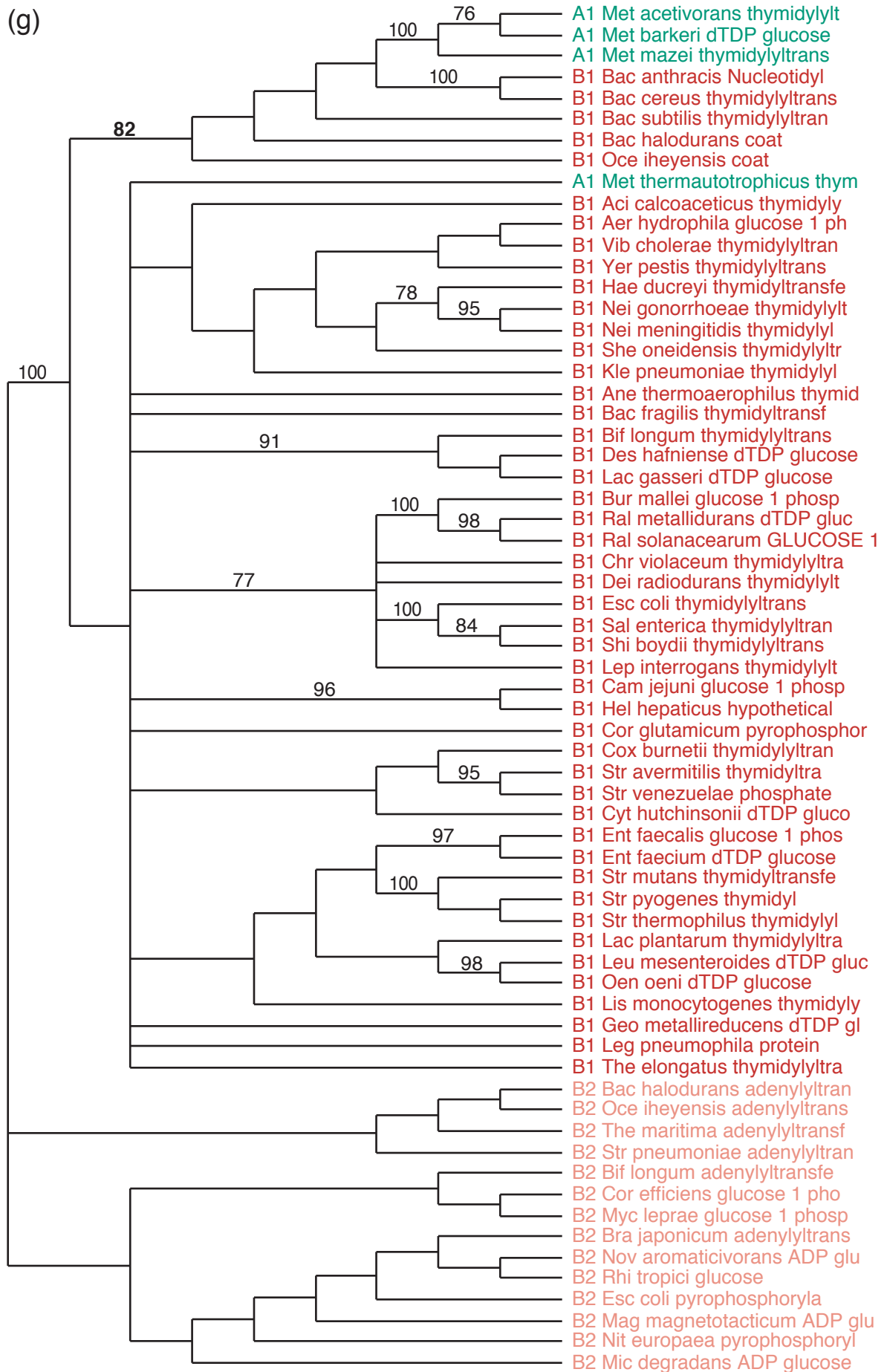

(h)

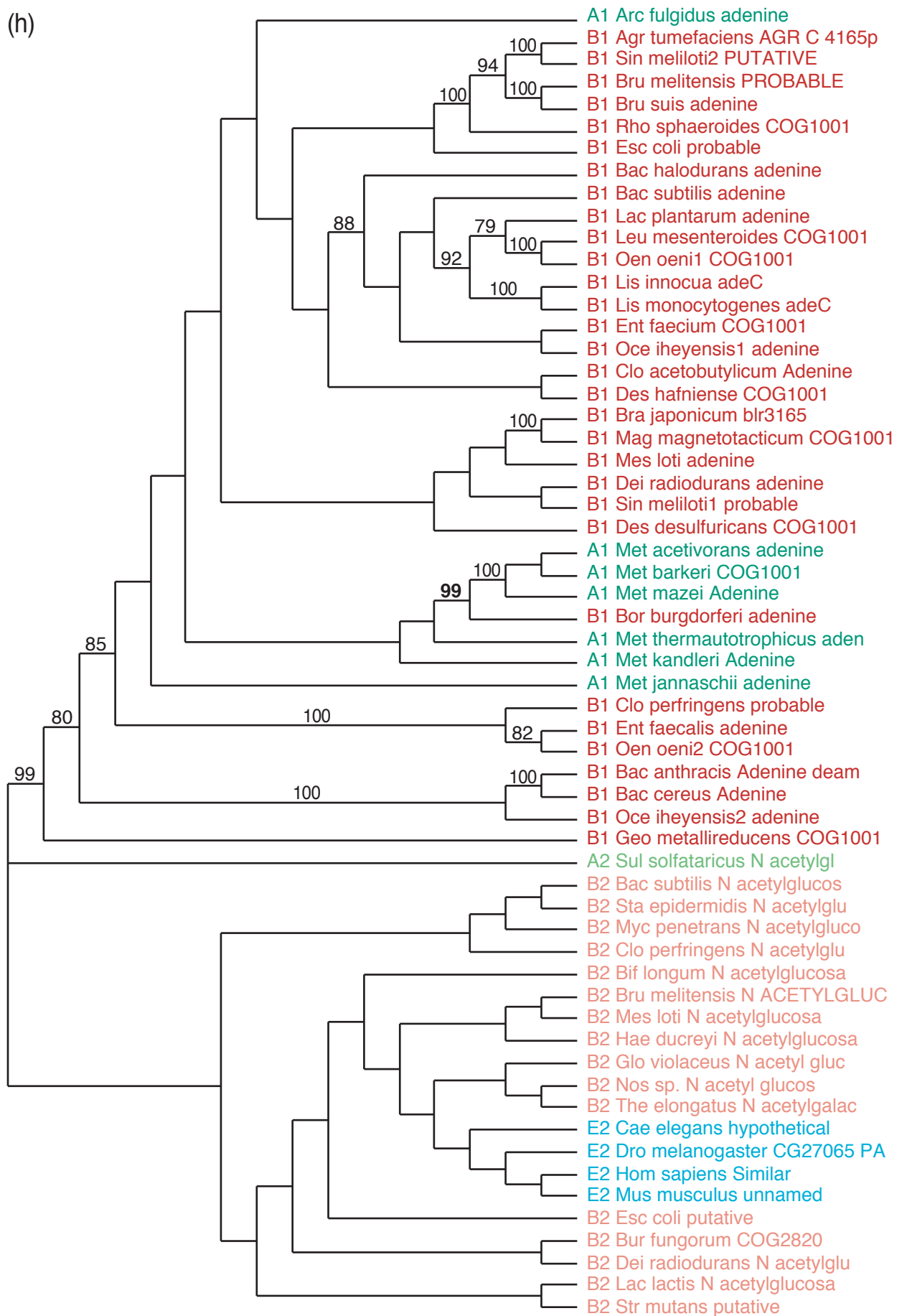

Supplement: File 3— — This file contains cladograms obtained for genes with phylogenetic evidence of IDHGT. Trees are strict consensus cladograms and numbers shown are bootstrap values above 75 (500 reps). Bootstrap values in bold font indicate possible HGT events. (a) 1-Deoxyxylulose-5-phosphate synthase (dxs, COG3959/3958), paralog Transketolase 2 isozyme; (b) Fructose-bisphosphate aldolase, class II (fba, COG0191), paralog Tagatose-bisphosphate aldolase 1; (c) Dipetide transport protein (dppa, COG0747), paralog Putative transport periplasmic protein; (d) ABC-type FE3+-siderophore transport system, permease component (HemU, COG0609), paralog ABC-type FE3+-siderophore transport system, ATP-binding; (e) Ferrous iron transport protein B (feoB, COG0370), paralog GTP-binding protein; (f) Formate dehydrogenase (fdhF, COG0243), paralog Nitrate reductase 1; (g) Glucose-1-phosphate thymidylyltransferase (rmla, COG1213), paralog Glucose-1-phosphate adenylyltransferase; (h) Adenine deaminase adeC, COG1001), paralog Putative N-acetylgalactosamine-6-phosphate deacetylase. To facilitate viewing the figures we have colored taxa from the three major domains as follows: Bacteria target genes (B1) are colored red; Bacteria paralog genes (B2) are colored orange; Archaea target genes A1) are colored green; Archaea paralog genes (A2) are colored lime; Eukarya target genes (E1) are colored blue; Eukarya paralog genes (E2) are colored aqua. [file ebo-4-109-s3.pdf]
